# Magnetic Resonance Imaging Findings of the Proximal Metacarpal Region in Warmblood Horses: 36 Lame and 26 Control Limbs (2015–2021)

**DOI:** 10.3389/fvets.2021.714423

**Published:** 2021-08-12

**Authors:** Elisabeth van Veggel, Kurt Selberg, Brenda van der Velde-Hoogelander, Nick Bolas, Katrien Vanderperren, Hendrik Jan Bergman

**Affiliations:** ^1^Sporthorse Medical Diagnostic Centre, Heesch, Netherlands; ^2^Department of Environmental and Radiological Health Sciences, College of Veterinary Medicine and Biomedical Sciences, Colorado State University, Fort Collins, CO, United States; ^3^Hallmarq Veterinary Imaging Ltd, Guildford, United Kingdom; ^4^Department of Medical Imaging of Domestic Animals and Orthopedics of Small Animals, Faculty of Veterinary Medicine, Ghent University, Merelbeke, Belgium

**Keywords:** MRI, equine, proximal suspensory ligament, forelimb, warmblood horse

## Abstract

**Objectives:** This study aims to evaluate the distribution and severity of bone and soft tissue lesions in the proximal metacarpal region of warmblood horses in lame and control groups. Correlation between lesions and ability to return to work was evaluated in the lame group.

**Methods:** This restrospective analysis evaluated 62 horses with MRI examination of the proximal metacarpal region between Sept 2015 and Feb 2021. There were 36 lame limbs and 26 control limbs. The control group included seven contralateral limbs.

**Results:** Proximal suspensory ligament (PSL) size was not different between the lame and control groups. Hyperintensity seen on T1W/T2^*^W GRE images within the dorsal collagenous part of the PSL and hyperintense Short-TI Inversion Recovery (STIR) signal within the dorsal collagenous part of the PSL or within the McIII were only present within the lame group. Palmar cortical McIII resorption and dorsal margin irregularity of the PSL and McIII sclerosis were more severe within the lame limbs, but mild gradations were also seen in control limbs. Intermediate gradings for a subset of lesions were commonly seen in the non-lame contralateral to lame limbs. Return to work in the lame group is not statistically different for any measured observation(s), and 19/33 of the lame horses returned to work at similar or higher levels.

**Conclusion and clinical importance:** Fifty-eight percent in this group of warmblood horses returned to work within a variable time frame. The majority (81%) of lame limbs showed bone and soft tissue abnormalities, but no enlargement of the PSL was noted in lame horses, and no correlation was seen between the severity or type of lesions and the ability to return to work. The presence of STIR hyperintensity within the proximal McIII or dorsal collagenous part of the PSL and hyperintensity within the dorsal collagenous part of the PSL on T1W GRE and T2^*^W GRE images, as well as significant palmar cortical McIII resorption are considered clinically relevant lesions. Contralateral limbs may not truly represent the normal condition, showing nonclinical variations and adaptive remodeling.

## Introduction

Proximal suspensory disease is an important cause of forelimb and hindlimb lameness in sport horses (1–4). Lameness associated with proximal suspensory disease can be variable depending on the lesion location and severity (2, 5, 6). The origin of the proximal suspensory ligament (PSL) in the forelimb has the main sagittal attachment located over the proximopalmar aspect of the third metacarpus (McIII), but there are also a few fibers originating from the palmar aspect of the third carpal bone just distal to the origin of the accessory ligament of the deep digital flexor tendon (ALDDFT) and from the axial aspect of the fourth metacarpal bone (7–10). The PSL is mainly made up of three tissues; collagenous fibers and, in the center of each lobe, striated muscle fibers and fat (7–10). The suspensory ligament is innervated by the deep branch of the palmar ramus of the ulnar nerve (11). Radiography and ultrasonography are frequently used for evaluation of the proximal metacarpal region. There are limitations associated with these techniques as lesions identified may not show a good correlation with the clinical lameness or may not be visualized ultrasonographically (7, 8, 12). Bone scintigraphy can be used if a bony component is part of the suspensory disease, but this technique is limited in the evaluation of the soft tissue component (6). MRI provides a more detailed information than ultrasonography and radiography and correlates morphologically well with histology (7). Furthermore, MRI may be a diagnostic aid when other modalities fail to identify clearly the cause of proximal metacarpal pain and may improve selection of adequate therapy and prognosis for injuries in this region (4, 10, 13, 14). Forelimb standing low-field MRI examinations were judged to be rewarding in the context of general case management as well as the detection of abnormalities (15).

The purpose of this study was to describe the MRI findings in a group of warmblood horses that presented for lameness associated with the proximal metacarpal region as well as a group of warmblood limbs that served as a control group. This was done to delineate which findings within the proximal metacarpus are clinically significant and which findings can be seen in non-lame limbs. Furthermore, this study aims to evaluate the correlation between type and severity of lesions and the rate and/or ability to return to work in warmblood horses.

Our hypothesis is that sclerosis of McIII and dorsal margin irregularity of the PSL can be seen in lame as well as sound horses, but that bony and/or soft tissue STIR hyperintensity, hyperintensity on T1W/T2^*^W GRE images within the dorsal collagenous part of the PSL, and bone resorption of the palmar aspect of McIII will only be observed in lame horses. In addition, it is hypothesized that horses with STIR hyperintensit*y* in the McIII were more likely to return to work than horses with soft tissue and/or enthesis injury.

## Materials and Methods

Medical records from the Sporthorse Medical Diagnostic Centre (SMDC) were reviewed from Sept 2015 to Feb 2021. Cases were included in retrospective analysis if they were warmblood horses and referred for an MRI of the proximal metacarpal region. For the lame group, only horses that were lame and blocked to either direct infiltration of the PSL or blocked *via* the lateral palmar nerve or high four-point nerve block with negative distal blocks were included. All lame horses were lame due to pain and did not have a neurologic or biomechanical component to their lameness. The control group contained 26 limbs which were separated into three subgroups. There were eight limbs that had no lameness anywhere and no abnormalities on clinical examination. There were seven limbs that had a contralateral limb lameness but had an MRI examination of both proximal metacarpal regions, and 11 limbs had a distal limb lameness localized with diagnostic anesthesia and significant distal limb MRI findings which were consistent with the clinical presentation. The control group showed no lameness arising from the proximal metacarpal region in the follow-up period and was separated into a true control (*n* = 19) and contralateral control group (*n* = 7). The true control group consisted of the eight limbs with no lameness and the 11 limbs where the lameness was located within the distal limb.

Lameness evaluation, diagnostic anesthesia, and conventional diagnostic imaging (ultrasonography and radiography) were done by the referring veterinarian when referred. Conventional diagnostic imaging did not provide a diagnosis for the lameness in all cases. The majority of the horses in the lame group were referral patients (21/36) for which conventional diagnostic imaging had been performed but was not consistently available for review. The lameness severity gradation was available for a subset of horses (20/36) with grades 1–3/5 recorded (*n* = 10 with grade 1, *n* = 7 with grade 2, and *n* = 3 with grade 3). These examinations were not repeated prior to the MRI, but horses were noted to be lame at the time of the MRI scan. All MRI scans were performed within 1 week of the diagnostic anesthesia.

MRI examinations were performed using a Hallmarq standing equine MRI with a 0.27-Tesla magnet. MRI of the proximal metacarpal region and grading was performed on a minimum of transverse T1W GRE, T2^*^W GRE, T2W FSE, and STIR FSE, as well as sagittal T1W GRE and frontal (dorsal) T1W GRE with additional scans used for interpretation if available.

**Figure 1:**
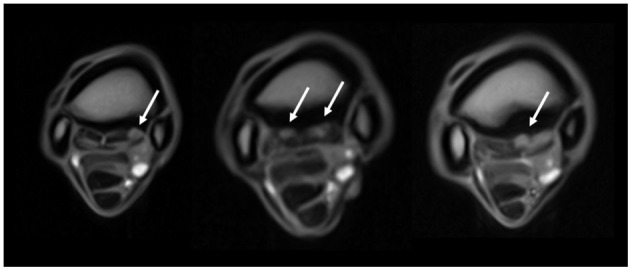
Grading of the hyperintensity within the dorsal collagenous part of the proximal suspensory ligament as seen on T1W GRE transverse images (from left to right, grades 1, 2, and 3 identified by the white arrows). The transverse images are 2–3 cm distal to the carpometacarpal joint, and all images are from limbs within the lame group.

**Figure 2:**
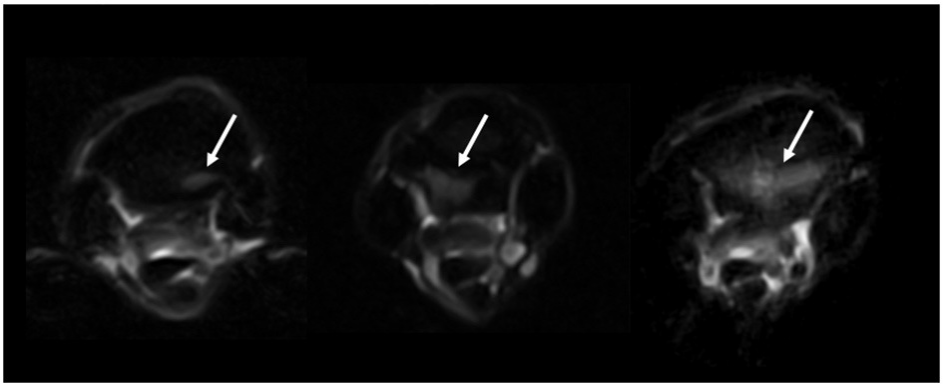
Grading of the STIR hyperintensity with the proximal metacarpus as seen on STIR FSE transverse images (from left to right, grades 1, 2, and 3 identified by the white arrows). The transverse images are located 1–2 cm distal to the carpometacarpal joint, and all images are from limbs within the lame group.

**Figure 3:**
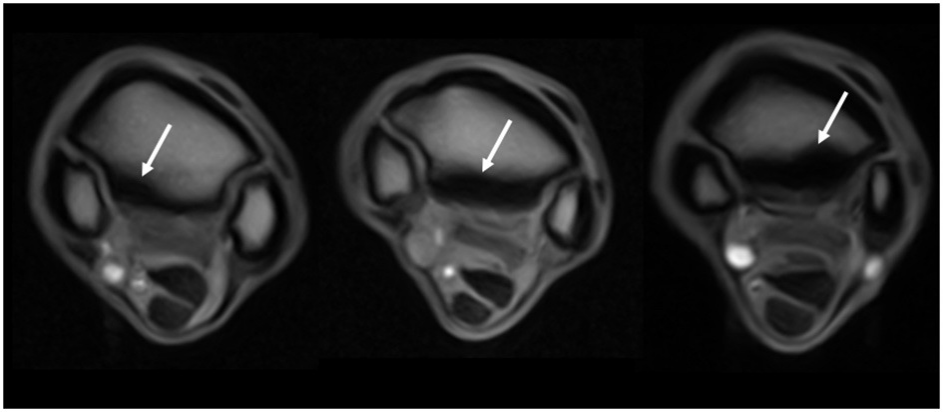
Grading of the sclerosis (abnormal low signal) of the proximal metacarpus as seen on T1W GRE transverse images (from left to right, grades 1, 2, and 3 identified by the white arrows). The transverse images are located 1–2 cm distal to the carpometacarpal joint. The left image is from a limb within the control group, and the middle and right images are from limbs within the lame group.

The images were evaluated by an ACVR board-certified radiologist (KS) prior to the study and were retrospectively graded again by the European College of Veterinary Diagnostic Imaging resident (EVV) while being blinded to the horse identity and group classification. Consensus was reached on all gradings of the images between KS and EVV. The grading scoring was modified from a previous high field study to suit the study using low field MRI (16) (Supplementary Table 1). The pathology was graded from 0 (absent) to 3 (severe), and distribution was noted for the following criteria: dorsal margin irregularity of the PSL, hyperintensity within the dorsal collagenous part of the PSL on T1W/T2^*^W GRE images, STIR hyperintensity within the dorsal collagenous part of the PSL, McIII STIR hyperintensity, McIII low signal (sclerosis), resorption of the palmar margin of McIII, osseous reaction of the McII/IV, and/or axial bone proliferation. Examples for gradation severities are provided in Figures 1–3 regarding hyper intensity within the dorsal collagenous part of the PSL, STIR hyperintensity within the proximal McIII and sclerosis of the proximal McIII respectively. Additionally, when available for interpretation, the full carpus was evaluated for the presence or absence of osteoarthritis and/or other bony changes (such as endosteal/trabecular low signal (sclerosis), endosteal/trabecular STIR hyperintensity or ligamentous changes) and their distribution.

The total transverse (cross-sectional) area of the PSL size was measured distal to its attachment to the McIII defined as position 2 previously (7). This is where the dorsal margin of the PSL became distinguishable from the palmar cortex of the McIII approximately 3 cm distal to the carpometacarpal joint (9). In addition to measuring the size of the PSL, a ratio was performed between the PSL and the McIII at the same level. This was done to evaluate if this would eliminate the variability of the PSL size based on the size of the limb. MRI analyses were performed using Osirix DICOM viewing software version 11, and measurements of the PSL and McIII at the same level were performed on T1W GRE images. MRI sequence parameters are listed within Supplementary Table 2.

Official show records were obtained from the FEI database to see if the horse returned to the sport successfully (meaning competing at or higher than previous level) with similar competition frequency. In the absence of an online database or if questions arose from the database, the owners or referring veterinarians were contacted to establish whether the horse returned to competition and/or were training at the same or higher level. The time from the date of the MRI to the next competition was noted as return to competition in days.

Statistical analysis was done using Python, pandas, and scipy.stats. To compare the continuous measures of PSL size, and PSL to McIII size ratio, between lame and control limbs, the unpaired *t*-test was used. For grade measures, non-parametric tests were used. For comparison of values, the Mann-Whitney *U* test was used. Where numbers were small, measures were divided into two groups and frequencies compared using Fisher's exact test. Grouping was most often between grade 0 and all other grades. To compare the frequencies of lesion occurrence in different regions, Fisher's exact test was again used where numbers were small, and the Chi-squared test where numbers were sufficiently large. Analysis for correlations between pathological findings was done using Fisher's exact test and Chi-squared tests.

## Results

A total of 62 limbs were evaluated of which 36 limbs were lame and blocked to the PSL region. Of the 36 lame limbs evaluated, there were 14 right front (RF) and 22 left front (LF) limbs. This group was represented by 23 showjumpers and 12 dressage horses and 1 horse used for driving. There were 12 mares, 13 geldings, and 11 stallions. The age ranged from 4 to 16 years with a mean age of 9 years.

In the control group, there were 18 RF and 8 LF limbs from 26 horses. This group was represented by 12 showjumpers, 11 dressage horses, and three horses used for recreational riding. There were five mares, 13 geldings, and eight stallions. The age ranged from 3 to 16 years with a median age of 9.5 years. Within the subdivision of the contralateral control group (*n* = 7), there were three LF and four RF limbs. This group was represented by six showjumpers and one dressage horse. There were four geldings and three stallions, and the age ranged from 6 to 13 years with a mean age of 10 years. Within the subdivision of the true control group (*n* = 19), there were five LF and 14 RF limbs. This group was represented by six showjumpers, 10 dressage horses, and three horses used for recreational riding. There were nine geldings, five mares, and five stallions, and the age ranged from 3 to 16 years of age with a mean age of 9.3 years.

Presence, severity, and location of MRI changes identified in the PSL and McIII are summarized in Tables 1–4, and values obtained with statistical analysis are listed in Supplementary Tables 3–5. Sclerosis of McIII was present in 89% of the limbs within the lame group, in 66% of the horses in the true control group, and 100% of the contralateral limb control group. Sclerosis of McIII was most often seen medially in all groups (81, 100, and 86 % in the lame, true control, and contralateral control groups, respectively). In 11/19 true control horses, there was mild McIII sclerosis and one horse had moderate McIII sclerosis. Within the contralateral limb control group, two had moderate sclerosis and one limb had marked sclerosis. Sclerosis of the McIII was significantly different in grade values between true control and lame groups (*p* < 0.05) but not in grade frequency (*p* > 0.05). The frequency of occurrence was significant (*p* < 0.05) when comparing a group comprising grades 0 (none) and 1 with a group comprising grades 2 and 3.

**Table 1:** Grading of pathological findings per limb in lame group identified using MRI.

**Grade**	**0 (normal)**	**1 (mild)**	**2 (moderate)**	**3 (severe)**
Dorsal margin irregularity of the PSL	6/36 (17%)	19/36 (53%)	10/36 (28%)	1/36 (3%)
Hyperintensity on T1W GRE and T2*W GRE within the dorsal collagenous part of the PSL	11/36 (31%)	10/36 (28%)	9/36 (25%)	6/36 (17%)
STIR signal within the dorsal collagenous part of the PSL	24/36 (67%)	7/36 (19%)	5/36 (14%)	0/36 (0%)
McIII STIR signal	12/36 (33%)	13/36 (36%)	6/36 (17%)	5/36 (14%)
McIII sclerosis	4/36 (11 %)	13/36 (36 %)	13/36 (36%)	6/36 (17%)
McIII palmar cortical resorption	12/36 (33 %)	9/36 (25 %)	13/36 (36 %)	2/36 (6%)
Axial bone proliferation of McIII	34/36 (94%)	0/36 (0%)	2/36 (6%)	0/36 (0%)
Osseous reaction of McII and McIV	35/36 (97%)	1/36 (3%)	0/36 (0%)	0/36 (0%)
Carpus osteoarthritis, STIR signal, and/or sclerosis	5/11 (45%)	2/11 (18%)	4/11 (36%)	0/11 (0%)

**Table 2:** Grading of pathological findings per limb in control group identified using MRI.

**Grade**	**Type of control limbs**	**0 (normal)**	**1 (mild)**	**2 (moderate)**	**3 (severe)**
Dorsal margin irregularity of the PSL	ALL	12/26 (46%)	11/26 (42%)	3/26 (12%)	0/26 (0%)
	TC	11/19 (58%)	7/19 (37%)	1/19 (5%)	0/19 (0%)
	CLAT	1/7 (14%)	4/7 (57%)	2/7 (29%)	0/7 (0%)
Hyperintensity on T1W GRE and T2*W GRE within the dorsal collagenous part of the PSL	ALL	26/26 (100%)	0/26 (0%)	0/26 (0%)	0/26 (0%)
STIR signal within the dorsal collagenous part of the PSL	ALL	26/26 (100%)	0/26 (0%)	0/26 (0%)	0/26 (0%)
McIII STIR signal	ALL	26/26 (100%)	0/26 (0%)	0/26 (0%)	0/26 (0%)
McIII sclerosis	ALL	7/26 (27%)	15/26 (58%)	3/26 (12 %)	1/26 (4%)
	TC	7/19 (37%)	11/19 (58%)	1/19 (5%)	0/19 (0%)
	CLAT	0/7 (0%)	4/7 (57%)	2/7 (29%)	1/7 (14%)
McIII palmar cortical resorption	ALL	22/26 (85%)	3/26 (12%)	1/26 (4%)	0/26 (0%)
	TC	17/19 (89%)	2/19 (11%)	0/19 (0%)	0/19 (0%)
	CLAT	5/7 (71%)	1/7 (14%)	1/7 (14%)	0/7 (0%)
Axial bone proliferation of McIII	ALL	24/26 (92%)	0/26 (0%)	2/26 (8%)	0/26 (0%)
Osseous reaction of McII and McIV	ALL	23/26 (88 %)	0/26 (0%)	3/26 (12%)	0/26 (0%)
Carpus osteoarthritis, STIR signal, and/or sclerosis	ALL	5/6 (83%)	1/6 (17%)	0/6 (0%)	0/6 (0%)

*Subdivisions between contralateral limbs (CLAT) and true control limbs (TC) were done for McIII sclerosis, palmar cortical resorption of the third metacarpus (McIII), and dorsal margin irregularity of the proximal suspensory ligament (PSL)*.

**Table 3:** Distribution of pathological findings in lame limbs.

**Finding**	**Number of cases**	**Medial**	**Lateral**	**Medial and lateral**	**Central**
McIII sclerosis	32/36	26/32 (81%)	1/32 (3%)	5/32 (16%)	–
McIII STIR signal	24/36	14/24 (58%)	5/24 (21%)	2/24 (8%)	3/24 (12.5%)
Hyperintensity on T1W GRE and T2*W GRE within the dorsal collagenous part of the PSL	25/36	19/25 (76%)	4/25 (16%)	1/25 (4%)	1/25 (4%)
STIR signal within the dorsal collagenous part of the PSL	12/36	7/12 (58%)	3/12 (25%)	1/12 (8%)	1/12 (8%)
McIII palmar cortical resorption	24/36	19/24 (79%)	3/24 (13%)	1/24 (4%)	1/24 (4%)
Dorsal margin irregularity of the PSL	30/36	23/30 (77%)	4/30 (13%)	2/30 (7%)	1/30 (3%)
Axial bone proliferation of McIII	2/36	2/2 (100%)	–	–	–
Osseous reaction of McII and McIV	1/36	1/1 (100%)	–	–	–

**Table 4:** Distribution of pathological findings in control limbs.

**Finding**		**Number of cases**	**Medial**	**Lateral**	**Medial and lateral**	**Central**
McIII sclerosis	ALL	19/26	18/19 (95%)	–	1/19 (5%)	–
	TC	12/19	12/12 (100%)	–	–	–
	CLAT	7/7	6/7 (86%)	–	1/7 (14%)	–
Dorsal margin irregularity of the PSL	ALL	14/26	9/14 (64%)	4/14 (29%)	1/14 (7%)	–
	TC	8/19	7/8 (87.5%)	–	1/8 (12.5%)	–
	CLAT	6/7	2/6 (33%)	4/6 (67%)	–	–
McIII palmar cortical resorption	ALL	4/26	4/4 (100%)	–	–	–
	TC	2/19	2/2 (100%)	–	–	–
	CLAT	2/7	2/2 (100%)	–	–	–
Axial bone proliferation of McIII	ALL	2/26	1/2 (50%)	1/2 (50%)	–	–
Osseous reaction of McII and McIV	ALL	3/26	2/3 (67%)	1/3 (33%)	–	–

*Subdivisions between contralateral limbs (CLAT) and true control limbs (TC) were done for McIII sclerosis, palmar cortical resorption of the third metacarpus (McIII) and dorsal margin irregularity of the proximal suspensory ligament (PSL)*.

Dorsal margin irregularity of the PSL was present in 83% of lame limbs, 54% of true control limbs, and 86% of contralateral control limbs. Mild dorsal margin irregularity of the PSL was present in 7/19 limbs in the true control group, but only one horse had a moderate classification. In the contralateral limb control group, six of seven limbs had dorsal margin irregularity and two of seven limbs had moderate gradation. Dorsal margin irregularity was statistically different (*p* < 0.05) between true control and lame limbs in both frequency and values of observed grades.

Resorption of the palmar cortical McIII was present in 67% of lame limbs, 11% true control limbs, and 29% contralateral control limbs. Within the lame group, there were nine limbs with grade 1, 13 limbs with grade 2, and two limbs with grade 3. Within the true control limbs, there were two limbs with grade 1. Within the contralateral, there was one limb with grade 1 and one limb with grade 2. Resorption of the palmar cortical McIII was significantly greater, in both number and severity, in lame horses than in all control horses (*p* < 0.05).

STIR hyperintensity within the proximal McIII was present in 67% of the horses in the lame group and was most often located medially (58%). Only one horse had STIR hyperintensity present within the proximal metacarpus without McIII sclerosis. Furthermore, within the lame group, eight of 36 horses had McIII sclerosis without STIR hyperintensity.

Hyperintensity within the dorsal collagenous part of the PSL on T1W and T2^*^W GRE images was present in 69% (25/36) of horses in the lame group with the majority located in the medial lobe (76%, 19/25).

When STIR hyperintensity within the dorsal collagenous part of the PSL was present, it was always associated with concurrent hyperintensity on T1W and T2^*^W GRE images (*p* < 0.05). However, only 48% of horses with T1W and T2^*^W GRE hyperintensity had STIR hyperintensity within the dorsal collagenous part of the PSL.

Osseous reaction of McII/IV was only present in one limb in the lame group and in three limbs in the control group. In the lame limb, there was STIR hyperintensity within the McIII as well hyperintensity on T1W and T2^*^W GRE scans within dorsal collagenous part of the PSL which correlated with the clinical presentation, and the osseous reaction of McII/IV had smooth margins with normal adjacent soft tissue and was therefore considered not clinically relevant. Axial bone proliferation from the McIII adjacent to the PSL was an uncommon finding, two of 36 and two of 26 in the lame and control group respectively and was present only medially in the lame group and medially or laterally in the control group.

Hyperintensity on T1W and T2^*^W GRE within the dorsal collagenous part of the PSL and presence of STIR hyperintensity in the dorsal collagenous part of the PSL or in the McIII were only noted to be present in the lame horses and thus significantly greater in lame horses (*p* < 0.05).

In 81% of the lame limbs, there was concurrent bone and soft tissue changes characterized by the presence of an alteration of the dorsal part of the PSL as well as palmar McIII pathology.

Concerning the carpus, there were 11 horses in which the entire carpus was scanned in addition to the PSL region. In seven of these horses, there were changes associated with the carpus and in two, the carpus was considered a component of the clinical lameness. The other five horses had minimal findings and the lameness was attributed solely to the changes in the proximal metacarpus. In the control group, there were six horses that underwent MRI examination of the entire carpus, of which one horse had a minor finding within the carpus.

For the lame group, the mean PSL CSA was 2.88 cm^2^ (min = 2.25 cm^2^, max = 3.723 cm^2^). For the control group (contralateral and true control combined), the mean PSL CSA was 2.81 cm^2^ (min = 2.22 cm^2^, max = 3.56 cm^2^, median = 2.77 cm^2^). The ratio of PSL to McIII was 21.9% (min = 17%, max = 27.7%) for the lame group and 21.7% (min = 17.2, max = 24.9%) for the control group. The size of the PSL as well as ratio of the PSL to the McIII at the same level were not significantly different between the two groups. When looking at discipline, there was no difference in the mean size of PSL between dressage and showjumpers (2.82 and 2.92 cm^2^, respectively, ratio 22.0 and 21.9%, respectively) in the lame group. For the control group, there was no significant difference in mean size of PSL between dressage and showjumpers (2.70 and 2.89 cm^2^, respectively, ratio 21.6 and 21.6%, respectively).

There was a statistical positive correlation between grade of McIII sclerosis and palmar cortical McIII resorption (*p* < 0.05). There was no significant relationship between dorsal margin irregularity of the PSL and hyperintensity on T1W and T2^*^W GRE images within the dorsal collagenous part of the PSL. Similarly, no significant relationship was seen between hyperintensity on T1W and T2^*^W GRE images within the dorsal collagenous part of the PSL and resorption of the palmar aspect of the McIII (*p* > 0.05).

Follow-up data was available for 33/36 of the lame horses. A total of 19/33 horses returned to work at the same or higher level, 10/33 horses did not return to work, three horses were still in rehab at the time of the study, and one horse died from colic. Within the group of 19 horses, one of the horses resumed full training at previous level but was not competed due to a non-orthopedic-related trauma. Of the horses that successfully returned to competition, there were 15 horses that were actively competing at an international level and three horses that returned to national competitions and/or home training. Of the 15 horses, the return to competition range was 26 to 440 days, average 249 days and median 220 days. There were six horses that returned to competition in 6 months or less, five horses in 12 months or less, and four horses that returned to competition 14 months or less. None of the horses in this study had surgery performed. Return to work in the lame group is not statistically different for any measured observation and multiple lesions do not affect ability to return to work (*p* > 0.05).

From the 19 horses that returned to work successfully, 14 limbs had STIR hyperintensity within the proximal McIII, 16 limbs had dorsal margin irregularity of the PSL, 13 limbs had hyperintensity within the dorsal collagenous part of the PSL on T1W and T2^*^W GRE images, six limbs had STIR hyperintensity within the dorsal collagenous part of the PSL, 18 limbs had McIII sclerosis, 13 limbs had resorption of the palmar cortical McIII, and one limb had osseous reaction of McII/IV.

From the 10 horses that did not return to work successfully, seven limbs had STIR hyperintensity within the proximal McIII, nine limbs had dorsal margin irregularity of the PSL, nine limbs had hyperintensity within the dorsal collagenous part of the PSL on T1W and T2^*^W GRE images, five limbs had STIR hyperintensity within the dorsal collagenous part of the PSL, eight limbs had McIII sclerosis, eight limbs had resorption of the palmar cortical McIII, one limb had osseous reaction of McII/IV, and one limb had axial bone proliferation of McIII medially.

## Discussion

In contrast to our hypothesis, return to work in the lame group is not statistically different for any measured observation(s). Having multiple lesions does not affect the ability to return to work. In agreement with our hypothesis, STIR hyperintensity in both the proximal McIII or the PSL as well as hyperintensity on T1W and T2W^*^ GRE images within the dorsal collagenous part of the PSL will only be seen in lame horses and palmar McIII resorption is significantly different between lame and control limbs. In contrast to our hypothesis, dorsal margin irregularity of the PSL and McIII sclerosis were significantly different between the lame and true control groups for gradation severity and for dorsal margin irregularity of the PSL also in frequency. However, mild dorsal margin irregularity of the PSL and McIII sclerosis can be observed in true control limbs as well as contralateral control limbs. Clinical implication of this finding is that mild McIII sclerosis and dorsal margin irregularity of the PSL can also be seen in horses that are not lame. Interestingly, contralateral limbs being represented by seven limbs were intermediate in grading for the dorsal margin irregularity of the PSL and McIII sclerosis and therefore may not considered true control limbs. With respect to palmar cortical resorption of the McIII, contralateral limbs were also intermediate in grading but were only represented by two cases. Similar to the true control group, no contralateral limbs had hyperintensity on T1 and T2^*^W images within the dorsal collagenous part of the PSL and hyperintense STIR signal within the dorsal collagenous part of the PSL or within the McIII. Patient-related work, conformation, and/or susceptibility to changes in the proximal metacarpal region may be related to the difference between contralateral and true control limbs.

As previously noted and as seen in this study, proximal metacarpal pain will likely have some degree of both bone and soft tissue involvement, and careful inspection of the dorsal margin of the PSL must be done (4, 16). In agreement with previous studies, nearly all horses have some degree of sclerosis in the palmar aspect of McIII more often located medially (9, 16). There was a statistically significant correlation between McIII sclerosis and palmar cortical McIII resorption. Sclerosis and resorption together may ultimately weaken the enthesis of the PSL and could play a critical role in the development of clinical disease (16). It would be interesting to follow-up on control horses over the years as well as follow-up on horses with McIII sclerosis without current palmar cortical McIII resorption. It is possible that control cases with mild resorption and moderate sclerosis will ultimately become clinical cases. Regarding the sclerosis, there is likely a continuum between adaptive remodeling and degenerative changes as sclerosis is present in 87.5% of lame horses and 66% of true control horses in this study (16–18). This continuum is further supported as lame horses had significantly increased lesion severity than control horses and contralateral limbs were intermediate in severity.

As previously reported, forelimbs were more likely to have pathology reported in the medial lobe or both lobes, the dorsal aspect of the margin, and the collagenous or muscle tissue (4). The fat muscle bundles were not specifically assessed in this study due to their considerable variation in the shape, distribution, and signal intensity (10, 12).

In a previous study, warmblood horses were found to have a mean PSL curved surface area (CSA) of 2.86 cm^2^ (min = 2.37 cm^2^, max = 3.59 cm^2^) (7). Our findings confirm a relatively large range of normal values and a similar mean and ranges to the previous study (7). There was no statistical difference between the two groups for PSL size. The large range of normal PSL sizes potentially makes measurement at a single timepoint ineffective, and serial measurements within an individual horse may offer more insight. To attempt to eliminate variation as warmblood horses may be quite variable in size, a ratio to the McIII was calculated. This continued to show a large variation (lame group 17–27.7% and control group 17.2–24.9%) confirming the variability in PSL size within warmblood horses. There was no significant difference between size of PSL when comparing showjumpers with dressage horses. Forelimb suspensory ligament does not seem to show the same tendency to enlarge considerably compared with hindlimb suspensory ligaments (5). This was confirmed considering the lack of difference between the size of the PSL in lame and control horses. This is in contrast to a recent publication with 21.5% of their cases having enlargement of the PSL including showjumpers and dressage horses (20 and 26%, respectively) (4).

Unfortunately, due to the large number of referral cases, we were not able to assess the possibe relationship between severity of lameness and MRI findings, nor correlate the findings with ultrasonography and/or radiography. However, it was previously noted that there is no significant correlation between the type or severity of lesion(s) and lameness (2, 5, 6, 16). Additionally, it is possible that clinically significant abnormalities can be present in the PSL that do not necessarily affect the linearity of the fibers, thereby creating relatively normal ultrasonography images but significantly abnormal magnetic resonance images (19).

The limited number of observations of osseous reaction of the splint bones including exostoses in our current study compared with previous studies may be due to the fact that horses with exostosis may not be referred for MRI and are followed up with ultrasound and/or radiographs or to a truly smaller number of these conditions (4, 16). Additionally, no fissures were present in the lame group of horses which is likely related to the fact that in our population of sport horses, these cases are often not blocked prior to examination and are sent for MRI based on severity of the clinical examination, negative lower limb blocking, and/or after scintigraphy. Since blocking to the PSL region was a criterion to be included in this study, fissures were therefore less likely to be included.

The majority (20/21 horses) of the limbs with McIII STIR hyperintensity had concurrent varying degrees of McIII sclerosis indicating trabecular remodeling. This is contrary to previous results where STIR hyperintensity was not always associated with trabecular remodeling (20). Interestingly, the only horse without McIII sclerosis had centrally located STIR hyperintensity and not a lateral or medial distribution.

Dorsal margin irregularity of the PSL was present in all evaluated groups although significantly more in number and severity in the lame limb group. Irregularity of the margins of the PSL, especially dorsally at 3 cm distal to the carpometacarpal joint, is often seen in normal horses (10). Irregularity of the margins of the PSL in low field GRE images should be interpreted with care as this irregularity was often not noted on high field images (21). This discrepancy was attributed to volume averaging, and factors such as lower spatial resolution and/or lower signal-to-noise ratio may also play a role.

Hyperintensity within dorsal collagenous part of the PSL on T1W and T2^*^W GRE images may represent degeneration, tearing, and/or fibrosis. A proportion of these horses also had corresponding STIR hyperintensity. There was no correlation between palmar McIII resorption and hyperintensity within the dorsal margin of the PSL on T1W and T2^*^W GRE images nor between hyperintensity within the dorsal margin of the PSL on T1W and T2^*^W GRE images and dorsal margin irregularity of the PSL. This lack of correlation between hyperintensity on T1 and T2^*^W and dorsal margin irregularity of the PSL confirms that these two lesions are likely to represent different disease processes.

In our study, 58% of lame group horses returned to competition with an additional three horses (representing 9%) still in rehabilitation and one (representing 3%) which did not return for other reasons. This finding was considerably lower than a previous study where 73% returned to competition irrespective of severity of injury (16). This may be related to the relatively older age of warmbloods (mean 9 years) present in our study.

Previous studies have indicated a generally favorable prognosis with acute proximal suspensory desmitis (5, 13). Only a very small number of the horses in our lameness group had sole desmitis of the PSL. The majority of lame limbs (81%) had concurrent suspensory ligament and palmar McIII abnormalities as seen previously (4). No horses with proximal metacarpal pain had MR imaging abnormalities of the ALDDFT in our study, contrary to previous studies (13, 19). The lack of abnormalities of the ALDDFT was in line with recent findings (4, 17). The lack of injury to superficial digital flexor tendon, ALDDFT, and/or deep digital flexor tendon may reflect caseload or case selection as horses in our study all had conventional imaging including ultrasonography before MRI.

On average, our population of warmblood horses returned to competition in less than 6 months with a quite variable range [1–14.5 month(s)]. Owner's investment and dedication may have influenced whether a horse was given the opportunity as well as time needed to return to work.

Limitations of the study included the small number of horses thereby limiting the statistics. Furthermore, due to the retrospective nature and partial referral population of horses, available clinical data was limited thus lameness severity comparisons were not an objective of this study. Unfortunately, the contralateral non-lame limb was not examined in all horses because of financial and time constraints. The carpometacarpal joint was examined in all cases, and the distal row of the carpal bones was examined in the majority of the scans. However, to be noted as having the carpus examined, a full MRI examination of carpus had to be completed up to the distal radius physis. In the author's experience, showjumper and dressage horses suffer more commonly from clinically relevant PSL injury than carpal injury. Unfortunately, the full carpus was only scanned in a subsection of patients. Lesions within the carpus may have been missed when significant changes were present with the proximal metacarpus and the carpus was not requested to be scanned. It was noted that carpometacarpal osteoarthritis was more common than osteoarthritis of the middle carpal joint (27 vs. 11%, respectively) (16). The carpometacarpal joint would have been visualized in all horses when the proximal metacarpal region was scanned. The middle carpal joint and/or antebrachiocarpal joint would have been visualized in those cases with a complete carpus scan. In addition, pathology within the distal limb of horses in the lame group cannot be completely excluded but was considered less likely based on the diagnostic anesthesia including negative distal limb diagnostic anesthesia as well as the imaging findings of the proximal metacarpal region.

Measurements of the PSL may not have been exactly at the same location distal to the carpometacarpal joint due to slice positioning and slice thickness. This may affect the size of the PSL (and McIII) obtained but is a factor also encountered in clinical practice.

Although the control group was not lame at the time of the MRI and had no history of lameness related to the proximal metacarpal area according to the referring veterinarian and/or owner, a previous or subclinical injury could not be excluded.

There was no comparison with the true gold standard which would be the (histo)pathologic examination of the suspensory ligament as this was not applicable in our clinical study.

Proton density (PD) sequences were added to the standard imaging sequences during the study period but were therefore not available for all studies and thus PD sequences were not assessed as part of this study. In the population of horses where PD sequences were used, they confirmed findings noted on other sequences but did not add significantly different information.

In conclusion, STIR hyperintensity in the dorsal collagenous part of the PSL or McIII and hyperintensity on T1W and T2^*^W images of the dorsal collagenous part of the PSL are clinically relevant findings. Palmar cortical McIII resorption is rarely seen in control horses, and moderate gradations must be considered clinically relevant. Only 58% of horses returned to competition with a variable time frame. No enlargement of the PSL was noted in lame horses, and the majority of lame horses showed presence of bone and soft injuries.

## Data Availability Statement

The original contributions presented in the study are included in the article/Supplementary Material, further inquiries can be directed to the corresponding author/s.

## Ethics Statement

Ethical review and approval was not required for the animal study as this was a retrospective study and study patient were clinical patients of the Sporthorse Medical Diagnostic Centre. Written informed consent for participation was not obtained from the owners as part of this study specifically, as patient owners signed a consent procedure for research when patients were initially examined.

## Author Contributions

EV and HB designed the study. EV and KS performed the execution of the study (MRI analysis). NB analysed the data. All authors contributed to the data interpretation, manuscript preparation, contributed to the article, and approved the submitted version.

## Conflict of Interest

NB is employed by Hallmarq Veterinary Imaging Ltd. The remaining authors declare that the research was conducted in the absence of any commercial or financial relationships that could be construed as a potential conflict of interest.

## Publisher's Note

All claims expressed in this article are solely those of the authors and do not necessarily represent those of their affiliated organizations, or those of the publisher, the editors and the reviewers. Any product that may be evaluated in this article, or claim that may be made by its manufacturer, is not guaranteed or endorsed by the publisher.

## References

[1] Denoix JM Perrot P Bousseau B Sciciuna C. Images échographiques des lesions du muscle interosseoux III (ligament suspenseur du boulet). Prat Vét Equine. (1991) 23:23–33.

[2] Gibson KT Steel CM. Conditions of the suspensory ligament causing lameness in horses. Equine Vet Educ. (2002) 14:39–50. 10.1111/j.2042-3292.2002.tb00137.x

[3] Meehan L Labens R. Diagnosing desmitis of the origin of the suspensory ligament. Equine Vet Educ. (2016) 28:335–43. 10.1111/eve.1233116881834

[4] Murray RC Tranquille CA Walker VA Milmine RC Bak L Tacey JB. Magnetic resonance imaging findings in the proximal metacarpal region of 359 horses and proximal metatarsal region of 64 horses acquired under standing sedation. J Equine Vet Sci. (2020) 94:103268. 10.1016/j.jevs.2020.103268 33077090

[5] Dyson SJ. Proximal metacarpal and metatarsal pain: a diagnostic challenge. Equine Vet. Educ. (2003) 15:134–8. 10.1111/j.2042-3292.2003.tb00231.x16509170

[6] Dyson SJ. Diagnosis and management of common suspensory lesions in the forelimbs and hindlimbs of sport horses. Clin Tech Equine Pract. (2007) 6:179–88. 10.1053/j.ctep.2007.08.004

[7] Bischofberger AS Konar M Ohlert S Geyer H Lang J Ueltschi G. Magnetic resonance imaging, ultrasonography and histology of the suspensory ligament origin: a comparative study of normal anatomy of Warmblood horses. Equine Vet J. (2006) 38:508–16. 10.2746/042516406X156109 17124840

[8] Denoix JM Coudry V Jacquet S. Ultrasonographic procedure for a complete examination of the proximal third interosseous muscle (proximal suspensory ligament) in the equine forelimbs. Equine Vet Educ. (2008) 20:148–53. 10.2746/095777308X282381

[9] Nagy A Dyson S. Magnetic resonance anatomy of the proximal metacarpal region of the horse described from images acquired from low- and high-field magnets. Vet Radiol Ultrasound. (2009) 50:595–605. 10.1111/j.1740-8261.2009.01589.x 19999342

[10] Nagy A Dyson S. Magnetic resonance imaging and histological findings in the proximal aspect of the suspensory ligament of forelimbs in nonlame horses. Equine Vet J. (2012) 44:43–50. 10.1111/j.2042-3306.2011.00365.x 21649714

[11] Barone R. Anatomie Compare des Mammifères Domestiques. Barone R, editor. Tome second Arthologie et Myologie, Editions Vigot, Paris. (1989).

[12] Werpy NM Denoix JM McIlwraith CW Frisbie DD. Comparison between standard ultrasonography, angle contrast ultrasonography, and magnetic resonance imaging characteristics of the normal equine proximal suspensory ligament. Vet Radiol Ultrasound. (2013). 54:536–47. 10.1111/vru.12051 23718137

[13] Brokken MT Schneider RK Sampson SN Tucker RL Gavin PR Ho CP. Magnetic resonance imaging features of proximal metacarpal and metatarsal injuries in the horse. Vet Radiol Ultrasound. (2007) 48:507–17. 10.1111/j.1740-8261.2007.00288.x 18018721

[14] Powell SE Ramzan PH Head MJ Shepherd MC Baldwin GI Steven WN. Standing magnetic resonance imaging detection of bone marrow oedema-type signal pattern associated with subcarpal pain in 8 racehorses: a prospective study. Equine Vet J. (2010) 42:10–7. 10.2746/042516409X471467 20121907

[15] Labens R Schramme MC Murray RC Bolas N. Standing low-field MRI of the equine proximal metacarpal/metatarsal region is considered useful for diagnosing primary bone pathology and makes a positive contribution to case management: a prospective survey study. Vet Radiol Ultrasound. (2020) 61:197–205. 10.1111/vru.12824 31800146

[16] Barrett MF Manchon PT Hersman J Kawcak CE. Magnetic resonance imaging findings of the proximal metacarpus in Quarter Horses used for cutting: retrospective analysis of 32 horses 2009-2012. Equine Vet J. (2018) 50:172–8. 10.1111/evj.12746 28833365

[17] Nagy A Dyson S. Magnetic resonance imaging findings in the carpus and proximal metacarpal region of 50 lame horses. Equine Vet J. (2012) 44:163–8. 10.1111/j.2042-3306.2011.00422.x 21895751

[18] Murray RC Vedi S Birch HL Lakhani KH Goodship AE. Subchondral bone thickness, hardness and remodelling are in?uenced by short-term exercise in a site-specific manner. J Orthop Res. (2001) 19:1035–42. 10.1016/S0736-0266(01)00027-4 11781002

[19] Werpy NM Denoix JM. Imaging of the equine proximal suspensory ligament. The Veterinary Clinics of North America Equine Practice. (2012) 28:507–25. 10.1016/j.cveq.2012.08.005 23177129

[20] Sampson SN Tucker RL. Magnetic resonance imaging of the proximal metacarpal and metatarsal regions. Clin Tech Equine Pract. (2007) 6:78–85. 10.1053/j.ctep.2006.11.007

[21] Werpy N. Magnetic resonance imaging of the equine patient: a comparison of high-and low-field system. Clin Tech Equine Pract. (2006) 6:37–45. 10.1053/j.ctep.2006.11.004

