# Supplementary material for: Magnetic Resonance Imaging Findings of the Proximal Metacarpal Region in Warmblood Horses: 36 Lame and 26 Control Limbs (2015–2021)
Source: Front Vet Sci. 2021 Aug 12;8:714423. doi: 10.3389/fvets.2021.714423 (PMC8388851; doi:10.3389/fvets.2021.714423)
Supplement: Supplementary file 1 [file Table_1.DOCX]

**Supplementary Information**

Supplementary table 1: The subjective MRI grading criteria used for the bone and soft tissue categories for low field (0.27T) standing MRI images.

| **Grade** | **0 (normal)** | **1 (mild)** | **2 (moderate)** | **3 (severe)** |
| --- | --- | --- | --- | --- |
| Dorsal margin Irregularity of PSL | Normal | Mild irregularity one lobe | Moderate irregularity one lobe or mild irregularity both lobes | Marked irregularity affecting one lobe or moderate to marked irregularity both lobes |
| Hyperintensity on T1W GRE and T2*W GRE within the tendious part of the PSL dorsally | Normal | Focal region | Multifocal regions or diffuse affecting <30 % of the PSL | Diffuse affecting >30 % of the PSL |
| PSL STIR signal | Normal | Mild increase signal focal regions | Moderate increase signal focal regions or mild diffuse <30% of the PSL | Diffuse increase affecting >30% of the PSL |
| McIII STIR signal | Normal | Mild regional | Moderate regional or mild diffuse signal | Marked regional or moderate or marked diffuse |
| McIII Sclerosis | Normal | Mild sclerosis regionally | Mild sclerosis diffusely or moderate sclerosis regionally | Marked sclerosis regionally or moderate to marked sclerosis diffusely |
| McIII Palmar Cortical Resorption | Normal | Mild focal resorption | Moderate focal resorption to mild diffuse resorption | Marked focal resorption to moderate to marked diffuse resorption |
| Axial bone proliferation of McIII | Normal | Mild | Moderate | Marked |
| Osseous reaction of McII and McIV | Normal | Mild osseous proliferation of axial aspect of splint bones or mild change of the interosseous ligaments | Moderate osseous proliferation of axial aspect of splint bones or moderate change of the interosseous ligaments | Severe osseous proliferation of axial aspect of splint bones or severe change of the interosseous ligaments |
| Carpus osteoarthritis, STIR signal, and/or sclerosis | Normal | Mild osteophyte formation, mild STIR signal or mild sclerosis | Moderate osteophyte formation, moderate STIR signal or moderate sclerosis | Marked osteophyte formation, marked STIR signal or marked sclerosis |

Supplementary Table 2 : Standing low field MRI parameters used during this study.

| Sequence | TR (ms) | TE (ms) | Flip angle (deg.) | FOV (cm) | Matrix Size | Slice thickness (mm) | Gap (mm) | FrxPh |
| --- | --- | --- | --- | --- | --- | --- | --- | --- |
| T1W GRE | 52 | 8 | 50 | 170 x 170 | 256 x 256 | 5 | 1 | 170x170 |
| T2*W GRE | 68 | 13 | 28 | 170 x 170 | 256 x 256 | 5 | 1 | 170x160 |
| T2W FSE | 1500 | 110 | 90 | 170 x 170 | 256 x 256 | 5 | 1 | 128x144 |
| STIR FSE | 1834 | 22 | 90 | 170 x 170 | 256 x 256 | 5 | 1 | 128x144 |

TR: repetition time. TE: echo time FOV: field of view. FrxPh = number of frequency encoding steps and number phase encoding steps.

Supplementary Table 3. p values when comparing the control ggroup (combined ‘true control’ and ‘contralateral’) with the lame group for the parameters assessed.

| **Parameter Evaluated** | **Unpaired t test** | **Fisher’s Exact test** | **Mann Whitney U test** |
| --- | --- | --- | --- |
| Size PSL (CSA) | 0.682 | x | x |
| Size PSL ratio with McIII (CSA) | 0.544 | x | x |
| Dorsal margin irregularity of the PSL | x | 0.005 | 0.001 |
| Hyperintensity on T1W GRE and T2*W GRE within the dorsal collagenous part of the PSL | x | <0.001 | <0.001 |
| STIR signal within the dorsal collagenous part of the PSL | x | 0.005 | 0.003 |
| McIII STIR signal | x | <0.001 | <0.001 |
| McIII Sclerosis | x | 0.067 | <0.001 |
| McIII Palmar Cortical Resorption | x | 0.000 | <0.001 |

Supplementary Table 4. p values when comparing the ability to return to work vs no return to work in the lame group of horses.

| **Parameter Evaluated** | **Fisher’s Exact test** | **Mann Whitney U test** |
| --- | --- | --- |
| Dorsal margin irregularity of the PSL | 1.000 | 0.364 |
| Hyperintensity on T1W GRE and T2*W GRE within the dorsal collagenous part of the PSL | 0.215 | 0.091 |
| STIR signal within the dorsal collagenous part of the PSL | 0.696 | 0.265 |
| McIII STIR signal | 1.000 | 0.666 |
| McIII Sclerosis | 0.537 | 0.195 |
| McIII Palmar Cortical Resorption | x | x |

Supplementary Table 5. p values of correlations between various parameters

|  | **Fisher’s Exact Test** | **Chi-squared test** | **Linear Regression** |
| --- | --- | --- | --- |
| Correlation between hyperintensity on T1W GRE and T2*W GRE within the dorsal collagenous part of the PSL & STIR signal within the dorsal collagenous part of the PSL | 0.001 | <0.001 | <0.001 |
| Correlation between dorsal margin irregularity and Hyperintensity on T1W GRE and T2*W GRE within the dorsal collagenous part of the PSL | 0.279 | x | x |
| Correlation between McIII sclerosis and palmar cortical resorption of the McIII | 0.001 | <0.001 | <0.001 |
| Multiple conditions leading to worse probability to return to work | 0.466 | x | x |
